# Point-of-Care Ultrasound of Upper Airway Changes During Cardiopulmonary Bypass in Coronary Artery Bypass Grafting: A Randomized Controlled Trial

**DOI:** 10.3390/medicina62101829

**Published:** 2026-09-22

**Authors:** Engin Çetin, Bedirhan Günel, Mehmet Yılmaz, Çağrı Düzyol, Merve Yazıcı Kara, Ahmet Yüksek, Evrim Duman, Fatihhan Zeytun

**Affiliations:** 1 Department of Anesthesiology and Reanimation, Kocaeli City Hospital, 41000 Kocaeli, Türkiye; 2 Department of Cardiovascular Surgery, Kocaeli City Hospital, 41000 Kocaeli, Türkiye; 3 Pulmonology Clinic, Balıkesir Atatürk City Hospital, 10100 Balıkesir, Türkiye

**Keywords:** cardiopulmonary bypass, endotracheal tube cuff pressure, upper airway soft tissue, point-of-care ultrasonography, coronary artery bypass grafting, difficult extubation

## Abstract

*Background and Objectives*: Elevated endotracheal tube (ETT) cuff pressure impairs tracheal mucosal perfusion and may contribute to upper airway soft-tissue changes. During cardiopulmonary bypass (CPB), the cuff usually remains inflated although ventilation is suspended, yet the relevance of cuff pressure management during CPB is unclear. We compared two ETT cuff pressure strategies during CPB (0 vs. 20–30 cmH_2_O) on point-of-care ultrasound (POCUS) measurements of upper airway soft tissue in patients undergoing coronary artery bypass grafting (CABG). *Materials and Methods*: In this single-center, prospective, randomized, observer-blinded trial, 76 patients (ASA III–IV, aged 18–80 years) undergoing elective CABG were randomized 1:1 to a 0 cmH_2_O or 20–30 cmH_2_O ETT cuff pressure strategy throughout CPB (n = 38 per group). Transesophageal echocardiography (TEE) was not used in any patient. POCUS measurements of lateral pharyngeal wall (LPW) thickness, tongue thickness, parapharyngeal distance, midsagittal tongue area, and tongue width were obtained at four time points (T0–T3). The primary analysis was the omnibus group × time interaction for LPW thickness in a generalized estimating equations (GEE) model; the between-group difference in change from T0 to T2 was a planned supporting contrast. *Results*: All 76 randomized patients completed the study and were analyzed. The group × time interaction for LPW thickness was significant (*p* < 0.001); the between-group difference in the increase in LPW thickness from T0 to T2 was 1.50 mm (95% CI 1.27 to 1.73), and 1.00 mm (95% CI 0.82 to 1.18) at postoperative hour 2. Parapharyngeal distance and tongue thickness showed the same pattern. No multiplicity-adjusted between-group difference in LPW thickness was detected at T0 or T1, and a sensitivity analysis using the pre-intervention time point (T1) as baseline gave consistent results (T2 vs. T1: 1.43 mm, 95% CI 1.30 to 1.57). In an exploratory, unadjusted secondary analysis, difficult extubation occurred in 0/38 vs. 7/38 patients (absolute risk difference 18.4 percentage points, 95% CI 5.4 to 33.4; *p* = 0.012). *Conclusions*: Complete cuff deflation during CPB was associated with smaller ultrasonographic upper airway soft-tissue changes after CPB. The trial was not powered for clinical outcomes; the lower frequency of difficult extubation is hypothesis-generating, and these data do not support a change in routine practice before confirmation in trials with patient-centered endpoints.

## 1. Introduction

The endotracheal tube (ETT) cuff, when inflated, exerts continuous mechanical pressure on the tracheal mucosa, potentially impairing capillary perfusion and predisposing to ischemic injury and local edema. Elevated cuff pressures (>25–30 cmH_2_O) have been associated with tracheal and laryngopharyngeal mucosal congestion and may contribute to upper airway edema [1,2,3]. In cardiac surgery, postoperative pulmonary dysfunction, upper airway edema, difficult extubation, and post-extubation stridor remain clinically significant complications [4,5].

During coronary artery bypass grafting (CABG), cardiopulmonary bypass (CPB) is characterized by non-pulsatile perfusion, hypothermia, and a pronounced inflammatory response that increases capillary permeability and interstitial fluid accumulation [6]. Mechanical ventilation is suspended during CPB, yet the ETT cuff usually remains inflated, so mechanical compression of the tracheal mucosa continues throughout bypass [2,7]. In this setting, persistent cuff pressure may further impair local venous and lymphatic drainage and contribute to upper airway edema [6].

Because gas exchange is maintained by the extracorporeal circuit, temporary cuff deflation during CPB is feasible without affecting oxygenation. Nevertheless, evidence on cuff pressure management during CPB remains limited, and randomized controlled data are scarce [7,8,9].

Point-of-care ultrasonography (POCUS) is a non-invasive, reproducible modality for assessing upper airway anatomy [10,11,12,13]. Lateral pharyngeal wall (LPW) thickness, tongue thickness, and parapharyngeal distance are ultrasonographic parameters that have been associated with post-extubation airway edema and difficult extubation [5,14,15]. These ultrasonographic parameters describe anatomical dimensions; they do not directly measure tissue water content and cannot by themselves establish the presence of edema.

Few randomized studies have examined cuff pressure management during CPB using ultrasonographic measurements of upper airway soft tissue. We therefore compared maintaining ETT cuff pressure at 0 cmH_2_O versus 20–30 cmH_2_O during CPB in patients undergoing CABG. The primary outcome was the ultrasonographic change in LPW thickness; secondary outcomes included time-dependent changes in other ultrasonographic parameters and postoperative respiratory outcomes, most notably difficult extubation. We hypothesized that a 20–30 cmH_2_O strategy would be associated with more pronounced ultrasonographic upper airway soft-tissue changes and potentially more frequent difficult extubation than a 0 cmH_2_O strategy.

## 2. Materials and Methods

### 2.1. Study Design and Setting

This single-center, prospective, randomized, controlled clinical trial with blinding of participants and outcome assessors (registered as double-blind) was conducted at a tertiary-care cardiac surgery center. Patient recruitment commenced on 24 September 2025 and was completed on 19 April 2026. Patients and the public were not involved in the design, conduct, or reporting of the study. The study was reported in accordance with the CONSORT 2025 guidelines [16]. Ethical approval was obtained from the Kocaeli University Clinical Research Ethics Committee (approval no. KAEK/11.bI.09; date of approval 12 June 2025), and the trial was prospectively registered at ClinicalTrials.gov (NCT07148024; registered 21 August 2025) before enrollment of the first participant. Written informed consent was obtained from all participants, and the study was conducted in accordance with the principles of the Declaration of Helsinki. No changes were made to the primary or secondary outcomes after trial registration.

### 2.2. Primary Outcome

The primary outcome was the ultrasonographic change in lateral pharyngeal wall (LPW) thickness across the four predefined time points (T0–T3). The primary hypothesis, corresponding to the registered primary outcome, was that the trajectory of LPW thickness over T0–T3 differs between cuff pressure strategies. Accordingly, the primary statistical test was the omnibus group × time interaction in the generalized estimating equations (GEE) model. The trial registration (ClinicalTrials.gov NCT07148024; Supplementary Material Table S7) defines the primary outcome as the change in LPW thickness across these four time points but does not specify the statistical model. The T2 interaction coefficient, i.e., the between-group difference in change from T0 to T2 with its 95% confidence interval, is reported as a planned supporting contrast to aid clinical interpretation; it is not the same estimand as the omnibus test. Between-group comparisons at the other time points and comparisons of T2–T0 change scores (Section 3.1) are supporting analyses.

### 2.3. Secondary Outcomes

Secondary outcomes were defined to evaluate the broader effects of different ETT cuff pressure management strategies during cardiopulmonary bypass on upper airway structures. Time-dependent ultrasonographic changes in parapharyngeal distance, tongue thickness, tongue width, and midsagittal tongue cross-sectional area were analyzed. In addition, the frequencies of postoperative respiratory events, including difficult extubation, stridor, oxygen desaturation, and reintubation requirement, were compared between the two groups. Difficult extubation was defined a priori, as a study-specific operational definition, as the need for airway-support maneuvers (jaw-thrust and/or insertion of an oral or nasopharyngeal airway) immediately after extubation because of inadequate upper airway patency; this definition is intended to capture early upper airway obstruction and is not equivalent to established definitions of extubation failure. Stridor was defined as a high-pitched inspiratory sound accompanied by increased respiratory effort following extubation. Oxygen desaturation was defined as SpO_2_ < 90% despite supplemental oxygen at 5 L/min via face mask. Stridor, desaturation, and reintubation were recorded by the intensive care team from extubation until discharge from the intensive care unit. Clinical secondary outcomes were not adjusted for multiplicity and are regarded as exploratory.

### 2.4. Participants

Patients aged 18–80 years with American Society of Anesthesiologists (ASA) physical status III–IV, a body mass index (BMI) ≤ 35 kg/m^2^, and scheduled for elective CABG surgery were eligible for inclusion. Exclusion criteria comprised a history of difficult intubation [17,18], anatomical upper airway abnormalities, acute upper respiratory tract infection, high risk of obstructive sleep apnea (STOP-Bang score > 3) [19], left ventricular ejection fraction < 30%, chronic kidney disease (estimated glomerular filtration rate < 30 mL/min/1.73 m^2^), and emergency surgery.

Patients with a BMI > 35 kg/m^2^ were excluded because submental and lateral-neck ultrasound windows deteriorate with increasing neck adiposity and because obesity independently alters pharyngeal soft-tissue dimensions, which would have introduced a confounder unrelated to cuff pressure. Emergency procedures were excluded because the standardized anesthetic, cuff-management, and four-time-point ultrasound protocol could not be guaranteed in these settings. These criteria yield a selected population (see Limitations).

### 2.5. Randomization and Blinding

Patients were randomly assigned in a 1:1 ratio using a computer-generated randomization sequence (random.org). The allocation sequence was generated by an independent anesthesiologist who was not involved in patient care and was concealed in sealed opaque envelopes. Group allocation was disclosed only to the anesthesia team responsible for implementing the cuff pressure intervention. The investigator performing ultrasonographic measurements and the statistical analysis team remained blinded to group assignment. Owing to the nature of the intervention, the surgical and intraoperative anesthesia teams could not be blinded.

### 2.6. Anesthesia Protocol

All patients underwent a standardized anesthetic induction consisting of propofol (1–2 mg/kg), fentanyl (2–5 μg/kg), and rocuronium (0.6 mg/kg). Drug dosages were titrated according to clinical response to maintain hemodynamic stability. Anesthesia was maintained with sevoflurane (0.7–1 minimum alveolar concentration [MAC]) and remifentanil infusion. Volume-controlled mechanical ventilation was applied using a tidal volume of 6–8 mL/kg ideal body weight and a positive end-expiratory pressure (PEEP) of 5 cmH_2_O. During cardiopulmonary bypass, mild hypothermia (34 °C) was targeted, and antegrade blood cardioplegia was used.

All patients were intubated with the same single-use polyvinyl chloride oral/nasal cuffed endotracheal tube with a high-volume, low-pressure cuff (Chilecom; Ar-Es Tıbbi, Türkiye). The internal diameter was 8.0 mm in men and 7.5 mm in women. Intubation was performed by direct laryngoscopy with a Macintosh blade; all patients were intubated on the first attempt, and no traumatic intubation (blood on the tube or blade, visible mucosal injury) was documented. Cuff pressure was set to 20–30 cmH_2_O immediately after intubation in all patients. Cormack–Lehane grade and initial cuff inflation volume were not recorded. Cardiopulmonary bypass was conducted according to a uniform institutional protocol with mild hypothermia (34 °C): the circuit was primed with 1000 mL of balanced crystalloid (Isolyte S, pH 7.4) and 200 mL of 20% mannitol (total prime volume 1200 mL), non-pulsatile pump flow was targeted at 2.4 L/min/m^2^ body surface area, and mean arterial pressure was maintained at 60–80 mmHg. Nadir hematocrit, transfusion volume, ultrafiltration volume, and central venous pressure during CPB were not recorded at the patient level, whereas vasoactive drug use was recorded. In the intensive care unit, both groups received the same fast-track protocol: propofol sedation until rewarming, fentanyl analgesia, sugammadex reversal of rocuronium, core temperature ≥ 36 °C before extubation, and a 30° head-up position (extubation criteria and timing are described below). Cumulative opioid and sedative doses, the exact train-of-four ratio and body temperature at extubation, and the exact interval from ICU admission to extubation were not recorded.

Transesophageal echocardiography (TEE) was not utilized in any patient to eliminate potential confounding effects related to TEE probe-induced pharyngeal compression and alterations in ETT cuff pressure [20]. As mechanical ventilation was routinely suspended during CPB, gas exchange was maintained entirely through the bypass circuit oxygenator during this period. Consequently, temporary deflation of the ETT cuff in the 0 cmH_2_O group did not create an additional ventilation leak problem. To minimize aspiration risk, an orogastric tube was inserted in all patients before CPB [9]. At the end of CPB, before the resumption of mechanical ventilation, cuff pressure was adjusted to 20–30 cmH_2_O using a cuff manometer in both groups. Patients were extubated according to the institutional intensive care unit protocol once extubation criteria were met, including a train-of-four (TOF) ratio ≥0.9, adequate spontaneous respiratory effort, and hemodynamic stability. Extubation was performed in the intensive care unit at approximately the sixth postoperative hour by the intensive care team, who were not involved in the intraoperative cuff pressure intervention and remained blinded to group allocation. Difficult extubation was recorded by this team according to the predefined criteria specified under Secondary Outcomes.

### 2.7. Intervention Protocol

Baseline ultrasonographic measurements (T_0_) were obtained before induction of anesthesia, and the T_1_ assessment was performed after tracheal intubation while cuff pressure was standardized to 20–30 cmH_2_O in both groups, before the initiation of cardiopulmonary bypass. At the start of CPB, once mechanical ventilation was suspended and gas exchange was maintained through the extracorporeal circuit, the ETT cuff in the 0 cmH_2_O group was completely deflated using a cuff manometer, resulting in a cuff pressure of 0 cmH_2_O [8], whereas in the 20–30 cmH_2_O group the cuff pressure was maintained between 20 and 30 cmH_2_O and monitored every 5 min using a cuff manometer, with all measurements recorded [7,8]. The assigned cuff pressure strategy was applied throughout the entire CPB period. At the conclusion of CPB, before the resumption of mechanical ventilation, cuff pressure was standardized to 20–30 cmH_2_O in both groups.

In both groups, cuff pressure was measured with the cuff manometer every 5 min throughout CPB: in the 20–30 cmH_2_O group it was adjusted to the target range whenever necessary, and in the 0 cmH_2_O group the reading confirmed that the cuff remained fully deflated. To minimize the risk of aspiration during cuff deflation, an orogastric tube was placed after induction while the cuff was inflated, the oropharynx was suctioned immediately before cuff deflation, and repeat oropharyngeal suctioning was performed before the cuff was re-inflated at the end of CPB. Patients were supine with the head in a neutral position. Continuous gastric decompression and tracheal suctioning before resumption of mechanical ventilation were not part of the protocol, and silent microaspiration was not assessed objectively.

#### Ultrasonographic Assessment

All ultrasonographic examinations were performed by a certified anesthesiologist with experience in more than 150 airway ultrasonography examinations who remained blinded to group allocation. A 7–15 MHz linear probe and a 5–8 MHz convex probe (LOGIQ P9, GE Healthcare, Chicago, IL, USA) were used. Measurements were obtained according to previously validated methodologies [14,15,21].

Lateral pharyngeal wall (LPW) thickness and parapharyngeal distance were assessed using a linear transducer positioned over the cervical region and directed toward the external acoustic meatus. After identification of the internal carotid artery (ICA) with color Doppler imaging, the lateral pharyngeal wall was visualized as a hyperechoic line. LPW thickness was defined as the perpendicular distance between the ICA and the pharyngeal mucosal surface, whereas parapharyngeal distance was measured as the perpendicular distance between the lateral border of the ICA and the skin surface.

Tongue-related measurements were obtained using a convex transducer placed in the submental region. Tongue width was measured in the transverse plane as the distance between the lateral margins of the tongue. For tongue thickness and midsagittal tongue cross-sectional area, the transducer was positioned in the midsagittal plane while patients remained in the supine position with the mouth closed. Tongue thickness was defined as the maximum vertical distance between the dorsal tongue surface and the submental skin. The midsagittal tongue cross-sectional area was determined by manually tracing the tongue contour on a frozen image, after which the area was calculated using the ultrasound system’s automated measurement software.

At each time point, every parameter was measured three times and the mean value was used for analysis. All measurements were obtained at end-expiration using standardized ultrasound settings and were recorded at four predefined time points: T_0_ (before induction), T_1_ (after intubation, before CPB and before cuff deflation in the 0 cmH_2_O group), T_2_ (end of CPB, obtained immediately before cuff re-inflation to 20–30 cmH_2_O in the 0 cmH_2_O group and before resumption of mechanical ventilation), and T_3_ (postoperative hour 2).

Only the mean of the three measurements was recorded; the individual triplicate values were not retained, so intra-observer reproducibility could not be quantified retrospectively (see Limitations).

### 2.8. Statistical Analysis

All statistical analyses were performed using IBM SPSS Statistics for Windows, Version 27.0 (IBM Corp., Armonk, NY, USA). Descriptive statistics were presented as mean ± standard deviation, median (interquartile range [Q1–Q3]), or frequency (%), as appropriate. Data distribution was assessed using the Kolmogorov–Smirnov test.

Normally distributed independent continuous variables were analyzed using the independent-samples *t*-test and reported as mean ± standard deviation. Non-normally distributed continuous variables were analyzed using the Mann–Whitney U test and presented as median (Q1–Q3). Categorical variables were analyzed using Pearson’s chi-square test with Yates’ continuity correction or Fisher’s exact test, as appropriate. A two-sided *p*-value < 0.05 was considered statistically significant.

Repeated-measures data, including LPW thickness, tongue thickness, parapharyngeal distance, tongue width, and midsagittal tongue cross-sectional area, were analyzed using linear generalized estimating equations (GEE; normal distribution, identity link) with the patient as the cluster, fixed effects for group, time (T0–T3), and their interaction, an exchangeable working correlation matrix, and robust (Huber–White sandwich) standard errors. The exchangeable structure was chosen because the four measurements are not equally spaced in time. Estimated marginal means with 95% CIs, between-group differences at each time point, and the group × time interaction coefficients (β, 95% CI) are reported (Section 3.1 and Supplementary Tables S1 and S2). As sensitivity analyses, the models were re-fitted with first-order autoregressive, unstructured, and independence working correlation structures (Supplementary Table S3). Residuals were inspected graphically and with the Shapiro–Wilk test; inference relies on robust sandwich standard errors, which reduce reliance on correct specification of the working covariance structure and provide asymptotically robust inference. As a further sensitivity analysis, the models were re-fitted using the pre-intervention time point (T1) as baseline (T1–T3; Supplementary Table S6). Within-group changes between consecutive and baseline-referenced time points were examined with the Wilcoxon signed-rank test (Supplementary Table S7; supporting analyses). As further sensitivity analyses, the GEE models were re-fitted with Mallampati class × time or CPB duration × time added (Supplementary Table S8); post hoc, a group × CPB duration × time interaction and Spearman correlations between Δ(T2–T0) and CPB duration were examined (Supplementary Table S9; exploratory).

Multiplicity. The primary analysis is a single test (LPW group × time interaction) and was not adjusted. Within each ultrasonographic parameter, Bonferroni correction was applied to the pairwise contrasts of estimated marginal means for the group × time combinations, as implemented in the SPSS GEE estimated-marginal-means procedure, separately for each parameter’s model; no correction was applied across the five parameters in the original analysis; in the revised analysis, the four secondary omnibus group × time tests were additionally adjusted across parameters with the Holm procedure, the LPW omnibus test being the single confirmatory primary test, and time-point comparisons are regarded as supporting/exploratory. Secondary clinical outcomes were not adjusted for multiplicity and are exploratory.

Change scores (T2–T0) were compared with Welch’s *t*-test (difference in means with 95% CI) when normally distributed and with the Mann–Whitney U test when not; in the latter case, the between-group difference is reported as the Hodges–Lehmann estimate of the median difference with its distribution-free 95% CI. Absolute risk differences for binary outcomes are reported with Newcombe hybrid-score 95% CIs. Baseline balance is described with standardized mean differences (SMD; post hoc) rather than significance tests, with |SMD| > 0.1 taken to indicate imbalance. Analyses not planned in advance (absolute risk difference, SMDs, GEE sensitivity analyses, and the Mallampati distribution of difficult-extubation events, the Mallampati- and CPB-duration-adjusted GEE models, the Mallampati-stratified comparison of difficult extubation, and the CPB-duration interaction analyses) are labelled post hoc. Outcomes and analyses in relation to the registration (NCT07148024): the registration lists change in LPW thickness at T0–T3 as the primary outcome and tongue width, tongue thickness (entered under the heading “tongue volume”) and the fluid–ultrasound correlation as secondary outcomes; it does not specify the statistical model. The registration text refers to “five time points” but lists the four time points (T0–T3) used in this study. Parapharyngeal distance, midsagittal tongue area and the clinical outcomes (difficult extubation, stridor, oxygen desaturation, reintubation), as well as the exclusion criteria for high obstructive sleep apnea risk, low ejection fraction and chronic kidney disease, were planned before enrolment (ethics committee submission) but are not listed in the registration; the clinical outcomes are regarded as exploratory. Cuff pressures are given in the registration in mmHg; the manometer used reads in cmH_2_O, and cmH_2_O is used throughout this article. The registered exploratory correlation between intraoperative fluid volume and ultrasonographic parameters was not analyzed (see Limitations).

All randomized participants completed follow-up and were included in the final analysis. No missing ultrasonographic measurements occurred during the study. Because there were no missing data, the intention-to-treat and per-protocol populations were identical, and all randomized patients were included in the analysis.

### 2.9. Sample Size Calculation

Sample size estimation was performed using G*Power software version 3.1.9.7. Calculations were based on the lateral pharyngeal wall thickness before and after surgery reported in the reference study [14] (13.85 ± 4.41 mm before and 15.49 ± 5.65 mm after surgery, n = 89). Cohen’s d was calculated as the difference of means (1.64 mm) divided by the pooled standard deviation (5.07 mm), giving d ≈ 0.32. For a two-group design, f = d/2, giving f ≈ 0.16, which was entered into G*Power. The G*Power “ANOVA: repeated measures, within–between interaction” routine was used with two groups, four measurements, a two-sided α of 0.05, and 90% power (1 − β). The correlation among repeated measures (0.5) and the nonsphericity correction (ε = 1.0) were pragmatic planning assumptions (the G*Power defaults), chosen because no pilot data on the four-time-point correlation structure were available; ε = 1.0 assumes sphericity and is therefore not conservative. The observed mean pairwise correlation among the four LPW measurements in the present data was 0.66 (range 0.36–0.96), higher than the assumed 0.5, which favors power for within-subject effects. A further limitation is that d was derived from the pre-/post-operative change in a single cohort and does not directly represent a between-group difference in change over time; the design analyzed (within–between interaction) nevertheless corresponds to the primary GEE group × time interaction test. A total sample size of 68 patients (34 per group) was required; to account for an anticipated dropout rate of approximately 10%, the final target sample size was increased to 76 participants.

## 3. Results

### 3.1. Participant Flow and Baseline Characteristics

Between 24 September 2025 and 19 April 2026, a total of 81 patients were assessed for eligibility. Three patients were excluded because they met the exclusion criteria, and two patients declined participation before randomization. The remaining 76 patients were randomized and allocated to either the 0 cmH_2_O group (n = 38) or the 20–30 cmH_2_O group (n = 38) (Figure 1). All randomized patients were included in the analysis (intention-to-treat population).

Baseline demographic and clinical characteristics are presented in Table 1 together with standardized mean differences (SMD; Supplementary Table S4). Age, BMI, ASA physical status, ejection fraction, diabetes mellitus, and hypertension were similar between groups (|SMD| < 0.1). Male sex (SMD 0.27) and, in particular, Mallampati class were imbalanced despite randomization: Mallampati class I was present in 23.7% vs. 5.3% of patients (SMD 0.54) and class II in 42.1% vs. 60.5% (SMD −0.37) in the 0 and 20–30 cmH_2_O groups, respectively. Because ETT size was assigned by sex, 7.5 mm tubes were slightly more frequent in the 20–30 cmH_2_O group (19/38 vs. 14/38).

Perioperative data are summarized in Table 2. Adherence to the cuff-pressure protocol: in the 20–30 cmH_2_O group, the recorded cuff pressure during CPB was 24.7 ± 3.3 cmH_2_O (range 20–30), no reading outside the target range was recorded, and the number of adjustments per patient was not documented; in the 0 cmH_2_O group, all 5 min checks confirmed a fully deflated cuff (Supplementary Table S5). Vasoactive support was required in 12/38 (31.6%) vs. 22/38 (57.9%) patients (*p* = 0.037). In the exploratory, unadjusted analysis of clinical secondary outcomes, difficult extubation occurred in 0/38 patients in the 0 cmH_2_O group and 7/38 (18.4%) in the 20–30 cmH_2_O group (absolute risk difference 18.4 percentage points, 95% CI 5.4 to 33.4; Fisher’s exact *p* = 0.012; a relative risk is not estimable because no events occurred in the 0 cmH_2_O group). All seven events were managed with jaw thrust and/or an airway adjunct; one of the seven subsequently developed stridor with SpO_2_ < 92% despite face-mask oxygen at 5 L/min approximately 4 h after extubation and was re-intubated within 5 min; the other six did not develop stridor. One patient in the 0 cmH_2_O group, who had not had difficult extubation, was re-intubated for the same reason (stridor with SpO_2_ < 92% despite oxygen) approximately 4 h after extubation (re-intubation 1/38 vs. 1/38). Neither re-intubated patient reached the pre-defined desaturation threshold (SpO_2_ < 90%) before re-intubation. Post-extubation stridor occurred in 1/38 vs. 5/38 patients (*p* = 0.200) and oxygen desaturation in 2/38 vs. 5/38 (*p* = 0.430). Oxygen desaturation occurred in three of the seven patients. The need for bag-mask ventilation, non-invasive ventilation and the duration of airway-support maneuvers were not recorded. The seven patients were of Mallampati class I (n = 2), II (n = 3), III (n = 1), and IV (n = 1) (post hoc). In a post hoc analysis stratified by Mallampati class (I vs. II–IV), the between-group difference persisted (Cochran–Mantel–Haenszel χ^2^ = 10.5, *p* = 0.001; Supplementary Table S8). Because the trial was powered for an ultrasonographic endpoint and only seven events occurred, this observation is hypothesis-generating. No significant between-group differences were observed in heart rate, mean arterial pressure, SpO_2_, EtCO_2_, airway pressures, fluid balance, operative duration, CPB duration, or aortic cross-clamp time (all *p* > 0.05).

**Table 2:** Intraoperative and perioperative clinical variables according to cuff pressure strategy.

Parameter	Group 0 cmH_2_O	Group 20–30 cmH_2_O	*p*-Value
**Fluid balance and perioperative fluid variables**			
Total fluid intake (mL)	4087.6 ± 409.0	4095.5 ± 470.0	0.938 ^t^
CPB diuresis (mL)	501.3 ± 172.6	539.5 ± 189.9	0.362 ^t^
CPB fluid balance (mL)	2035 (1540–2100)	1935 (1515–2100)	0.722 ^m^
Blood loss (mL)	800 (672.5–902.5)	840 (630–922.5)	0.913 ^m^
**Hemodynamic variables**			
MAP before intubation (mmHg)	88.9 ± 10.1	88.5 ± 9.5	0.879 ^t^
HR before intubation (bpm)	79.2 ± 10.2	78.4 ± 11.7	0.731 ^t^
MAP after intubation (mmHg)	82.2 ± 10.7	81 ± 10	0.618 ^t^
HR after intubation (bpm)	78.8 ± 10.6	77.5 ± 13.4	0.656 ^t^
MAP at CPB separation (mmHg)	71.9 ± 6.6	71.6 ± 9.7	0.879 ^t^
HR at CPB separation (bpm)	85.8 ± 10.6	84.6 ± 13.8	0.689 ^t^
**Respiratory and airway-related variables**			
Peak pressure (cmH_2_O)	21 (19–22)	21 (20–22.3)	0.357 ^m^
Plateau pressure (cmH_2_O)	16 (15–18.3)	16 (14.8–19)	0.851 ^m^
SpO_2_ before intubation (%)	97 (97–98.3)	98 (97–98.3)	0.241 ^m^
SpO_2_ after intubation (%)	98 (98–99)	98 (98–99)	0.825 ^m^
EtCO_2_ after intubation (mmHg)	35 (32–37)	34 (32–35.3)	0.178 ^m^
SpO_2_ at CPB separation (%)	97 (96–98)	96 (95–98)	0.274 ^m^
EtCO_2_ at CPB separation (mmHg)	35 (34–37.5)	35.5 (33–39.3)	0.474 ^m^
Difficult extubation	0 (0)	7 (18.4)	**0.012 ^z^**
Stridor	1 (2.6)	5 (13.2)	0.200 ^z^
Oxygen desaturation	2 (5.3)	5 (13.2)	0.430 ^z^
Re-intubation	1 (2.6)	1 (2.6)	1.000 ^z^
Vasoactive support	12 (31.6)	22 (57.9)	0.037 ^z^
Cuff pressure during CPB (cmH_2_O)	0 (deflated)	24.7 ± 3.3 (20–30)	—
**Procedural variables**			
Operation duration (min)	212 (196.5–244.8)	203.5 (189.5–232.3)	0.237 ^m^
CPB duration (min)	98 (93.8–106.3)	99.5 (86.5–103.3)	0.509 ^m^
Cross-clamp duration (min)	61.5 (54–65.3)	63 (48.8–69.3)	0.674 ^m^

Data are presented as number (%), mean ± standard deviation, or median (Q1–Q3). ^z^ Fisher’s exact test; ^m^ Mann–Whitney U test; ^t^ independent-samples *t*-test. Total fluid intake comprises all crystalloid and colloid administered from induction to ICU admission, including circuit prime, cardioplegia, and blood products; CPB fluid balance is the net value recorded by the perfusionist for the CPB period (intake: prime, cardioplegia, added fluids and blood products; output: ultrafiltrate and urine during CPB); the individual components were not recorded separately, and urine output outside CPB is not included. Clinical outcomes (difficult extubation, stridor, oxygen desaturation, re-intubation) are exploratory and unadjusted for multiplicity; for difficult extubation, the absolute risk difference is 18.4 percentage points (95% CI 5.4 to 33.4). Vasoactive support: 12 (31.6%) vs. 22 (57.9%), *p* = 0.037 (Fisher’s exact test). CPB, cardiopulmonary bypass; EtCO_2_, end-tidal carbon dioxide; HR, heart rate; MAP, mean arterial pressure; SpO_2_, peripheral oxygen saturation.

Ultrasonographic measurements obtained at each time point are presented in Table 3 (descriptive values and supporting time-point comparisons); the primary omnibus test is reported in Table 4. Estimated marginal means with 95% CIs and between-group differences at each time point are given in Supplementary Table S1.

**Table 3:** Airway-related ultrasonographic parameters by group and time point: descriptive values and supporting time-point comparisons. The primary omnibus test (GEE group × time interaction for LPW thickness) is reported in Table 4.

Parameter/Time Point	Group 0 cmH_2_O	Group 20–30 cmH_2_O	Adjusted *p*-Value
**LPW** ** (mm)**			
T_0_	10.9 ± 0.4	11.0 ± 0.4	1.000
T_1_	11.2 ± 0.4	11.3 ± 0.5	1.000
T_2_	12.2 ± 0.5	13.8 ± 0.7	**<0.001**
T_3_	11.9 ± 0.5	12.9 ± 0.6	**<0.001**
**Tongue width (mm)**			
T_0_	48.1 ± 0.8	48.0 ± 1.0	1.000
T_1_	48.2 ± 0.9	48.3 ± 1.0	1.000
T_2_	48.4 ± 0.9	48.9 ± 1.1	1.000
T_3_	48.3 ± 0.9	48.7 ± 1.1	1.000
**Midsagittal tongue area (cm^2^)**			
T_0_	25.6 ± 1.2	25.8 ± 1.2	1.000
T_1_	25.3 ± 1.2	25.2 ± 1.2	1.000
T_2_	24.6 ± 1.4	23.8 ± 1.3	0.242
T_3_	24.8 ± 1.3	24.4 ± 1.2	1.000
**Tongue thickness (mm)**			
T_0_	55.8 ± 1.6	55.8 ± 1.2	1.000
T_1_	56.1 ± 1.6	56.8 ± 1.3	1.000
T_2_	56.9 ± 1.7	58.2 ± 1.4	**0.003**
T_3_	56.7 ± 1.6	57.8 ± 1.3	0.076
**Parapharyngeal (mm)**			
T_0_	15.1 ± 0.8	15.2 ± 0.9	1.000
T_1_	15.4 ± 0.9	15.3 ± 1.0	1.000
T_2_	16.2 ± 1.0	18.0 ± 1.3	**<0.001**
T_3_	15.9 ± 1.0	17.0 ± 1.1	**<0.001**

Data are presented as mean ± standard deviation. Bold *p*-values indicate *p* < 0.05. LPW, lateral pharyngeal wall. Adjusted *p*-values are Bonferroni-corrected pairwise comparisons of GEE estimated marginal means within each parameter. For LPW, these are supporting time-point comparisons; for the four secondary ultrasonographic parameters, they are exploratory follow-up comparisons. These *p*-values were not adjusted across parameters.

At T_1_, before the initiation of CPB and before cuff deflation in the 0 cmH_2_O group, no significant between-group differences were observed in any ultrasonographic parameter, including LPW thickness (11.2 ± 0.4 mm vs. 11.3 ± 0.5 mm; *p* = 1.000) and parapharyngeal distance (15.4 ± 0.9 mm vs. 15.3 ± 1.0 mm; *p* = 1.000), i.e., in multiplicity-adjusted cross-sectional comparisons these parameters did not differ between groups before the intervention. In these cross-sectional comparisons, between-group differences appeared after the CPB period (for interaction coefficients, including the small pre-intervention T1 interactions for the secondary parameters, see Supplementary Tables S2 and S6). At the end of CPB (T_2_), LPW thickness was significantly higher in the 20–30 cmH_2_O group than in the 0 cmH_2_O group (13.8 ± 0.7 mm vs. 12.2 ± 0.5 mm; *p* < 0.001), as was parapharyngeal distance (18.0 ± 1.3 mm vs. 16.2 ± 1.0 mm; *p* < 0.001). Tongue thickness differed significantly between the groups only at the T_2_ time point (58.2 ± 1.4 mm vs. 56.9 ± 1.7 mm; *p* = 0.003).

Primary analysis. The omnibus GEE group × time interaction for LPW thickness was significant (Table 4). The planned supporting contrast, the between-group difference in change from T0 to T2 (T2 interaction coefficient), was 1.50 mm (95% CI 1.27 to 1.73), and the corresponding difference at T3 was 1.00 mm (95% CI 0.82 to 1.18); there was no between-group difference at T0 (0.04 mm, 95% CI −0.13 to 0.21) or T1 (0.11 mm, 95% CI −0.09 to 0.31) (cross-sectional differences in estimated marginal means, Supplementary Table S1; the corresponding T1 interaction coefficient was 0.07 mm, 95% CI −0.06 to 0.19, Supplementary Table S2). Interaction coefficients with 95% CIs for all parameters are given in Supplementary Table S2, and estimated marginal means with between-group differences at each time point in Supplementary Table S1. The regression coefficients and robust standard errors were identical to the reported precision under exchangeable, autoregressive, unstructured, and independence working correlation structures (Supplementary Table S3), as expected for a saturated mean model with complete, balanced data. Adjustment for Mallampati class × time or for CPB duration × time did not materially change the interaction coefficients (LPW, T2: 1.49 mm, 95% CI 1.25 to 1.72, and 1.56 mm, 95% CI 1.33 to 1.78, respectively; Supplementary Table S8). Within-group sequential changes are given in Supplementary Table S7. In a post hoc analysis, the between-group difference in the T0→T2 change increased with CPB duration for parapharyngeal distance (0.29 mm per 10 min of CPB, 95% CI 0.10 to 0.47; *p* = 0.004) but not for LPW thickness (*p* = 0.682; Supplementary Table S9). The four secondary omnibus interactions remained significant after Holm adjustment across parameters (all adjusted *p* < 0.001). Interaction coefficients at T1 (before the intervention) were small but different from zero for the four secondary parameters (Supplementary Table S2); with T1 as baseline (Supplementary Table S6), the between-group differences in change to T2 were 1.43 mm (95% CI 1.30 to 1.57) for LPW thickness, 1.79 mm (1.58 to 2.01) for parapharyngeal distance, 0.70 mm (0.51 to 0.89) for tongue thickness, 0.39 mm (0.27 to 0.52) for tongue width, and −0.68 cm^2^ (−0.91 to −0.45) for midsagittal tongue area. The results of the GEE analysis for group, time, and group × time effects are summarized in Table 4; the group × time interaction was significant for all five parameters, most strongly for LPW thickness and parapharyngeal distance and less so for tongue width and midsagittal tongue area.

**Table 4:** Generalized estimating equations analysis of the effects of group, time, and group × time interaction on airway measurements.

Effect	Wald	df	*p*-Value
**LPW**			
Group	43.598	1	**<0.001**
Time	3747.063	3	**<0.001**
Group × Time	665.579	3	**<0.001**
**Tongue width**			
Group	1.058	1	0.304
Time	178.000	3	**<0.001**
Group × Time	42.162	3	**<0.001**
**Midsagittal tongue area**			
Group	1.207	1	0.272
Time	328.738	3	**<0.001**
Group × Time	37.931	3	**<0.001**
**Tongue thickness**			
Group	5.260	1	**0.022**
Time	603.546	3	**<0.001**
Group × Time	126.062	3	**<0.001**
**Parapharyngeal**			
Group	11.173	1	**<0.001**
Time	1416.505	3	**<0.001**
Group × Time	685.085	3	**<0.001**

Bold *p*-values indicate *p* < 0.05. df, degrees of freedom; LPW, lateral pharyngeal wall. The group × time interaction for LPW thickness is the single confirmatory primary test (unadjusted); the group × time *p*-values for the four secondary parameters are Holm-adjusted across parameters; group and time main-effect *p*-values are unadjusted and descriptive.

The Δ(T2–T0) analyses are supporting change-score analyses (Table 5): the LPW analysis supports the primary outcome, whereas the corresponding analyses of the other four parameters support the secondary ultrasonographic outcomes. The increase in LPW thickness was greater in the 20–30 cmH_2_O group than in the 0 cmH_2_O group (2.8 ± 0.6 mm vs. 1.3 ± 0.4 mm; difference in means 1.50 mm, 95% CI 1.26 to 1.74; *p* < 0.001), as was the increase in parapharyngeal distance and tongue thickness. The T2–T0 changes in tongue width and midsagittal tongue area also differed between groups (Table 5); for tongue width, which was not normally distributed, the between-group difference is given as the Hodges–Lehmann estimate of the median difference (0.60 mm, 95% CI 0.40 to 0.70).

**Table 5:** Comparison of T_2_–T_0_ changes in airway-related ultrasonographic parameters between study groups.

Parameter	Group 0 cmH_2_O (n = 38)	Group 20–30 cmH_2_O (n = 38)	95% CI	*p*-Value
ΔLPW T_2_–T_0_ (mm)	1.3 ± 0.4	2.8 ± 0.6	1.50 (1.26 to 1.74)	**<0.001 ^t^**
ΔTongue width T_2_–T_0_ (mm)	0.4 (0.1–0.5)	1.0 (0.6–1.1)	0.60 (0.40 to 0.70)	**<0.001 ^m^**
ΔMidsagittal area T_2_–T_0_ (cm^2^)	−1.0 ± 0.6	−2.0 ± 0.9	−0.99 (−1.33 to −0.66)	**<0.001 ^t^**
ΔTongue thickness T_2_–T_0_ (mm)	1.1 ± 0.5	2.4 ± 0.7	1.34 (1.06 to 1.63)	**<0.001 ^t^**
ΔParapharyngeal T_2_–T_0_ (mm)	1.1 ± 0.5	2.7 ± 0.9	1.61 (1.29 to 1.92)	**<0.001 ^t^**

Data are presented as mean ± standard deviation or median (Q1–Q3). ^m^ Mann–Whitney U test, with the between-group difference expressed as the Hodges–Lehmann estimate of the median difference and its distribution-free 95% CI; ^t^ Welch’s *t*-test, with the difference in means and its 95% CI. The LPW analysis is a supporting analysis of the primary outcome; the analyses of the remaining four parameters support the secondary ultrasonographic outcomes. The *p*-values in this table are unadjusted for multiplicity. Bold *p*-values indicate *p* < 0.05. LPW, lateral pharyngeal wall.

### 3.2. Harms

No cases of accidental extubation, clinically significant ventilatory leak, aspiration, or ventilation failure occurred during cardiopulmonary bypass in either group. Two patients (one in each group) required re-intubation in the intensive care unit approximately 4 h after extubation because of stridor with SpO_2_ < 92% despite face-mask oxygen at 5 L/min; both were re-intubated within 5 min of the onset of stridor and without complication. Furthermore, no serious adverse events attributable to the intervention were observed, and no participant required withdrawal from the study because of intervention-related complications.

## 4. Discussion

In this randomized, observer-blinded trial, complete deflation of the ETT cuff during cardiopulmonary bypass (0 cmH_2_O) was associated with smaller increases in ultrasonographic measurements of upper airway soft tissue than the standard strategy (20–30 cmH_2_O) in patients undergoing CABG. The primary analysis, the group × time interaction for lateral pharyngeal wall (LPW) thickness, was significant, with a between-group difference in change from T0 to T2 of 1.50 mm (95% CI 1.27 to 1.73). The evidence provided by this trial should be read at four distinct levels: (i) a randomized effect on ultrasonographic measurements; (ii) a possible physiological mechanism; (iii) an exploratory clinical signal regarding difficult extubation; and (iv) the absence of definitive evidence of an effect on extubation failure, reintubation, aspiration, pneumonia, or other patient-centered outcomes. These levels are addressed in turn below.

Our findings are consistent with previous ultrasonographic studies of the upper airway. Aytaç and Soyal reported increased LPW thickness and parapharyngeal distance after robotic prostatectomy, associated with intraoperative fluid loading [14], and Cetin et al. showed that higher pneumoperitoneum pressures during laparoscopic cholecystectomy produced more pronounced ultrasonographic upper airway changes [22]. The greater increases in LPW thickness and parapharyngeal distance in the higher-pressure group are compatible with cuff-related mechanical compression contributing to an increase in upper airway soft-tissue thickness during CPB. It should be emphasized that the measured parameters are anatomical dimensions: they do not quantify tissue water content or prove edema, and the observed changes may reflect a combination of tissue fluid accumulation, vascular filling, tissue geometry, muscle tone, patient position, and measurement conditions.

Elevated cuff pressure impairs tracheal mucosal capillary perfusion and reduces venous and lymphatic drainage [1,2,3], promoting congestion and interstitial fluid accumulation in the laryngopharyngeal region. This is biologically consistent with Seegobin and van Hasselt, who showed that elevated cuff pressures rapidly impair tracheal mucosal blood flow [2], and with the literature on post-extubation laryngeal edema [23].

For the primary outcome, LPW thickness, the groups did not differ at baseline (T0) or at T1, before cuff deflation, and the between-group difference emerged only after CPB (T2–T3). For the secondary parameters, this was not entirely the case: the group × time interaction coefficients at T1 were small but different from zero for parapharyngeal distance, tongue thickness, tongue width, and midsagittal tongue area (Supplementary Table S2), i.e., these parameters had already changed slightly differently between groups between T0 and T1, before the intervention began. The reason is unclear (chance imbalance in small pre-CPB changes is the most plausible explanation), but it means that the causal interpretation of the secondary ultrasonographic differences must be more cautious than for LPW thickness. In a sensitivity analysis using T1 as the baseline (Supplementary Table S6), the between-group differences in change from T1 to T2 and T3 remained significant and of similar magnitude for all parameters, which argues against the pre-intervention differences explaining the post-CPB findings. Because both arms underwent the same bypass procedure, and intraoperative fluid administration, diuresis, fluid balance, blood loss, CPB duration, and cross-clamp time were similar (Table 2), large systematic differences in CPB-related factors such as non-pulsatile perfusion, hemodilution, and inflammation are unlikely. Randomization does not, however, eliminate individual variability in inflammatory response, endothelial permeability, venous congestion, or hemodilution, and several CPB-related variables (nadir hematocrit, transfusion, ultrafiltration, central venous pressure) were not collected, so residual biological variability cannot be excluded. The exploratory observation that the between-group difference in parapharyngeal distance increased with CPB duration (Supplementary Table S9) is compatible with a cumulative, exposure-dependent effect of cuff pressure, but it was not pre-specified and requires confirmation. Vasoactive support was more frequent in the 20–30 cmH_2_O group; although this difference arose after randomization and is not plausibly caused by cuff management, vasoactive therapy and the hemodynamic instability that prompts it may themselves influence pharyngeal tissue dimensions. This pattern is consistent with a contribution of the cuff pressure strategy applied during bypass, although the precise mechanism cannot be established [2,7].

How might cuff pressure, which is not in direct contact with the pharynx, be associated with changes in the lateral pharyngeal wall and parapharyngeal region? These structures share loose submucosal connective tissue and venous and lymphatic drainage with the trachea and larynx [14,21], so impaired laryngopharyngeal outflow related to cuff pressure may be reflected in these adjacent, distensible tissues rather than at the cuff–mucosa interface [1,2]. A comparable indirect pattern has been described for pneumoperitoneum, where raised intra-abdominal pressure is associated with head-and-neck venous congestion despite no direct contact [14,22]. We selected the LPW and parapharyngeal region because they are reproducible ultrasonographic parameters that have been associated with post-extubation airway compromise in observational studies [5,14,15]. The greater increases at T2 and T3 in the higher-pressure group are consistent with CPB effects being amplified by continuous cuff-related compression [7,9]; this is a possible mechanism and remains a hypothesis.

Tongue width and midsagittal tongue area behaved differently from LPW thickness and parapharyngeal distance. The reduction in midsagittal tongue cross-sectional area, which contrasts with the increases observed in the pharyngeal parameters, is consistent with the findings of Aytaç and Soyal, who likewise reported a postoperative decrease in midsagittal tongue area alongside increases in lateral pharyngeal wall and parapharyngeal thickness [14]. A possible explanation is anesthesia-related loss of tongue muscle tone and geometric redistribution of the tongue, as also observed with pneumoperitoneum [14,22]; this interpretation is speculative and is not directly supported by the present data. Although the group × time interaction was statistically significant for tongue width and midsagittal tongue area, the magnitude of the effect was much smaller than for the pharyngeal parameters, and these two measurements may be better regarded as geometric parameters than as markers of soft-tissue thickening.

The 1.50 mm between-group difference in ΔLPW is statistically robust (95% CI 1.27 to 1.73). However, no prospectively validated minimal clinically important difference for LPW change exists, in cardiac surgical patients or otherwise; the associations reported in previous observational studies [5,15] should not be interpreted as a validated threshold, and the present study was not designed to define one. Statistical significance should therefore not be equated with established clinical importance. The absence of aspiration, significant air leak, unplanned extubation, or ventilation failure suggests that a carefully controlled 0 cmH_2_O strategy during CPB warrants further investigation in larger studies [7,8], but does not by itself establish safety (see Section 4.2).

### 4.1. Exploratory Clinical Observation: Difficult Extubation

The lower incidence of difficult extubation in the 0 cmH_2_O group (0/38 vs. 7/38; absolute risk difference 18.4 percentage points, 95% CI 5.4 to 33.4) is clinically interesting, but it rests on seven events in a trial not powered for this outcome, was not adjusted for multiplicity, and relies on a study-specific operational definition. The last ultrasonographic assessment (T3, postoperative hour 2) preceded extubation (approximately postoperative hour 6) by about four hours, so airway anatomy at the moment of extubation was not assessed, and no direct mechanistic link between the T3 findings and the extubation events can be inferred. In addition, Mallampati class was imbalanced at baseline, and unmeasured factors such as cumulative opioid and sedative exposure and the exact train-of-four ratio and body temperature at extubation could have contributed. The seven events were distributed across Mallampati classes I–IV, which argues against the baseline imbalance being the sole explanation, but with so few events, a contribution cannot be excluded. More generally, randomization in a relatively small sample does not guarantee balance in clinically relevant baseline airway characteristics, as the Mallampati distribution in this trial illustrates. The ultrasonographic between-group differences were essentially unchanged after adjustment for Mallampati class, and the difference in difficult extubation persisted in a Mallampati-stratified analysis (Supplementary Table S8), although both analyses are post hoc. This finding should be regarded as hypothesis-generating; it does not demonstrate that cuff deflation prevents difficult extubation. In particular, re-intubation did not differ between groups (1/38 vs. 1/38) and clinically relevant respiratory events were infrequent, so this observation should not be read as evidence of clinical efficacy.

### 4.2. Safety and Patient-Centered Outcomes

Complete cuff deflation removes the airtight seal during the period without mechanical ventilation. Although the absence of ventilation removes the need for a seal, it does not eliminate the possibility of aspiration of oropharyngeal or gastric contents, and an orogastric tube alone does not prevent microaspiration. Our protocol included oropharyngeal suctioning before deflation and before re-inflation, and no clinically detected aspiration occurred, but silent microaspiration was not assessed objectively. With one re-intubation in each group and no aspiration or pneumonia events, this trial cannot establish an effect on extubation failure, reintubation, aspiration, pneumonia, or other patient-centered outcomes, and the safety of the technique must be established before it can be recommended for wider clinical use.

A key strength is that all ultrasonographic measurements were performed in a standardized manner by a blinded investigator. In addition, no patient underwent transesophageal echocardiography (TEE), which can increase ETT cuff pressure and compress pharyngeal structures [20]; eliminating this confounder strengthens attribution of the observed effects to the cuff pressure intervention. At the same time, TEE is used routinely in many cardiac surgical centers, and the magnitude of the cuff-pressure effect observed here may differ when a TEE probe is in place; the deliberate exclusion of TEE therefore restricts the external validity of our findings to TEE-free CABG.

### 4.3. Limitations

This study has several important limitations. First, the single-center design and relatively modest sample size may limit the generalizability of the findings to broader and more heterogeneous patient populations. Second, upper airway changes were assessed exclusively with ultrasonographic measurements of anatomical dimensions, without validation against laryngoscopy, endoscopy, computed tomography, or magnetic resonance imaging; the measurements do not quantify tissue water content and cannot prove edema. Third, all ultrasonographic measurements were performed by a single investigator, and only the mean of triplicate readings was recorded; consequently, neither intra-observer nor inter-observer reproducibility could be quantified. Because the between-group differences are of the order of 1–2 mm, measurement precision is central to the interpretation of the results, and the lack of a formal reproducibility assessment is an important limitation. Fourth, we did not measure the tracheal air-column diameter, which would more directly reflect cuff-related changes at the cuff–mucosa interface; the lateral pharyngeal wall and parapharyngeal region were selected because they are reproducible ultrasonographic parameters associated with peripharyngeal soft-tissue changes, and future studies could combine these with air-column measurements. Moreover, the final ultrasonographic assessment was obtained at the second postoperative hour, whereas extubation was performed at approximately the sixth postoperative hour; consequently, airway status was not imaged at the time of extubation, and the temporal relationship between the observed ultrasonographic changes and difficult extubation cannot be established.

Several further limitations should be noted. Patients at high risk of obstructive sleep apnea, with known or anticipated difficult airways, with a BMI > 35 kg/m^2^, or undergoing emergency surgery were excluded, producing a selected, relatively low-risk airway population in which the benefit or risk of cuff deflation may differ. TEE was deliberately not used, which restricts external validity to TEE-free procedures. Several potentially relevant variables were not collected at the patient level: nadir hematocrit, transfusion and ultrafiltration volumes, central venous pressure, a complete perioperative net fluid balance up to the time of T3, cumulative opioid and sedative doses, the exact train-of-four ratio and temperature at extubation, the number of cuff-pressure adjustments, Cormack–Lehane grade, and initial cuff volume; these are potential contributors to postoperative upper airway changes and to the extubation events. The severity of the difficult-extubation events was not graded prospectively beyond the need for jaw thrust and/or an airway adjunct. Finally, although the five ultrasonographic parameters were planned in advance and analyzed as separate families, the number of secondary comparisons is large relative to the sample size, and nominally significant secondary results should be interpreted with caution.

In addition, the study did not compare complete cuff deflation with intermediate cuff pressure reduction strategies (e.g., 10–15 cmH_2_O). Consequently, it remains unclear whether the observed effect was specifically attributable to complete cuff deflation or to cuff pressure reduction in general. Although a difference in endotracheal tube distribution between groups could theoretically have influenced intubation conditions or cuff-related trauma, all patients underwent a standardized intubation protocol and no difficult intubations were reported. Silent microaspiration was not assessed using objective diagnostic methods. In addition, the registered exploratory relationship between intraoperative fluid volume and ultrasonographic airway changes was not formally analyzed; because fluid parameters were comparable between the groups, patient-level correlation analyses in larger cohorts are needed to address this question.

Furthermore, the study was powered for the primary ultrasonographic outcome of LPW thickness; therefore, it was underpowered for secondary clinical outcomes such as difficult extubation and stridor, and the clinical comparisons are exploratory. Finally, the findings are limited to patients undergoing CABG surgery. Because CPB duration and the magnitude of the inflammatory response may differ in valve surgery or combined cardiac procedures, direct extrapolation of these results to other cardiac surgical populations should be undertaken with caution.

## 5. Conclusions

In patients undergoing CABG surgery, complete deflation of the ETT cuff during cardiopulmonary bypass was associated with smaller ultrasonographic increases in lateral pharyngeal wall thickness, parapharyngeal distance, and tongue thickness than maintenance of 20–30 cmH_2_O. These findings support an association between cuff pressure management during CPB and ultrasonographic upper airway soft-tissue changes. The trial does not demonstrate that cuff deflation improves clinically meaningful airway outcomes, and the lower frequency of difficult extubation is an exploratory, hypothesis-generating signal. Cuff deflation during CPB should not be adopted as routine practice on the basis of these data. Because TEE was deliberately not used, these findings apply to TEE-free CABG and may not generalize to procedures in which a TEE probe is in place. Future trials should be powered for patient-centered endpoints—clinically important airway obstruction, stridor, extubation failure, reintubation, aspiration and microaspiration, postoperative pulmonary complications, and ICU length of stay—and should include an objective assessment of aspiration risk.

## Figures and Tables

**Figure 1:**
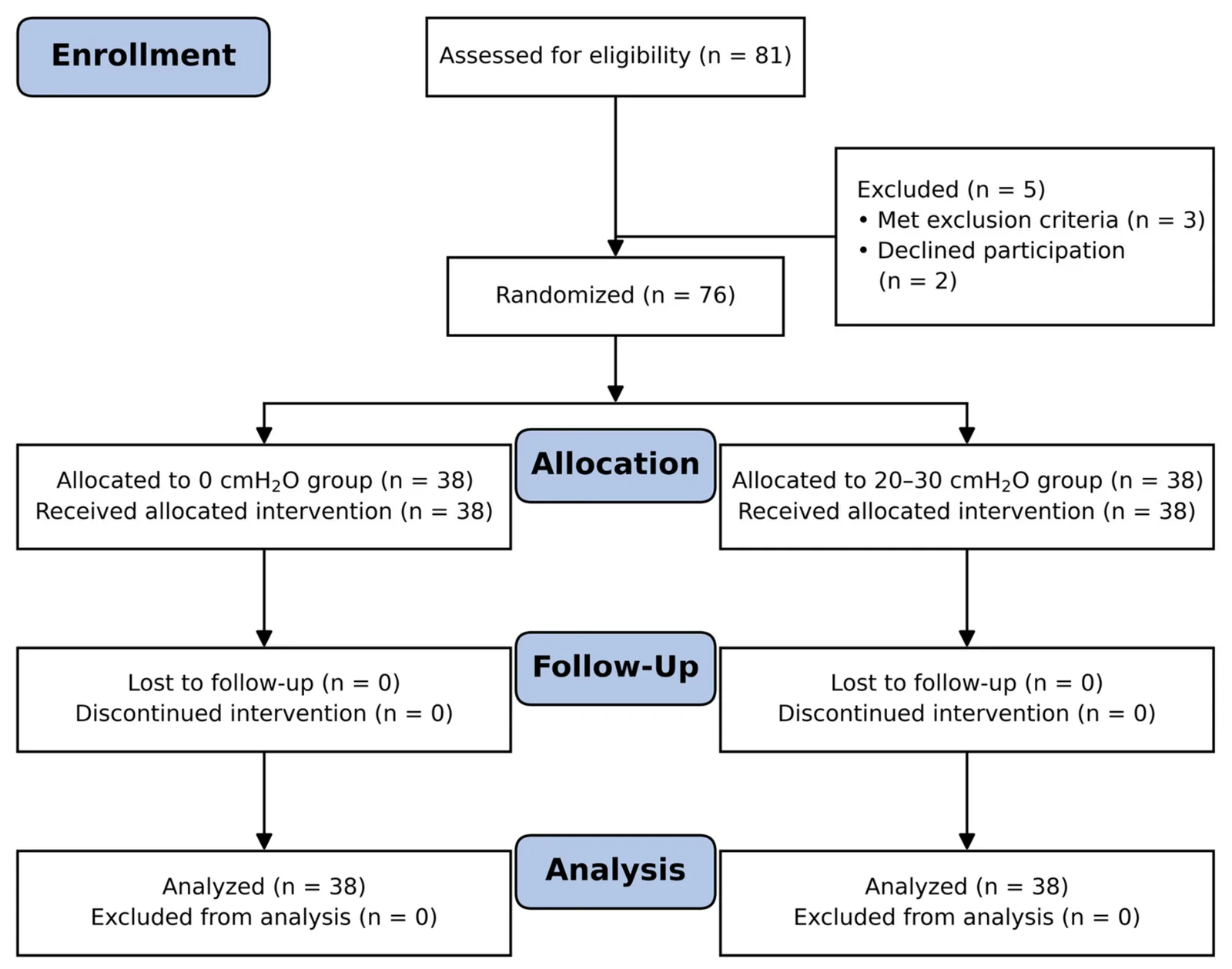
CONSORT flow diagram of patient enrollment and follow-up.

**Table 1:** Baseline demographic and clinical characteristics.

Characteristic	Group 0 cmH_2_O	Group 20–30 cmH_2_O	SMD
Age (years), median (Q1–Q3)	65 (58.8–71.3)	67 (60–71)	−0.07
BMI (kg/m^2^), mean ± SD	26.7 ± 2.6	26.8 ± 2.4	−0.05
Sex, n (%)			
Male	24 (63.2)	19 (50.0)	0.27
Female	14 (36.8)	19 (50.0)	−0.27
ASA classification, n (%)			
ASA III	30 (78.9)	31 (81.6)	−0.07
ASA IV	8 (21.1)	7 (18.4)	0.07
Mallampati score, n (%)			
Mallampati I	9 (23.7)	2 (5.3)	0.54
Mallampati II	16 (42.1)	23 (60.5)	−0.37
Mallampati III	10 (26.3)	11 (28.9)	−0.06
Mallampati IV	3 (7.9)	2 (5.3)	0.11
Ejection fraction (%), mean ± SD	47.2 ± 6.8	46.9 ± 7.1	−0.04
Diabetes mellitus, n (%)	26 (68.4)	25 (65.8)	−0.06
Hypertension, n (%)	32 (84.2)	31 (81.6)	−0.07

Data are presented as number (%), mean ± standard deviation (SD), or median (Q1–Q3), as indicated for each row. SMD, standardized mean difference (post hoc; 0 cmH_2_O minus 20–30 cmH_2_O); |SMD| > 0.1 indicates imbalance. ASA, American Society of Anesthesiologists; BMI, body mass index. Endotracheal tube internal diameter was 8.0 mm in men and 7.5 mm in women (8.0 mm: 24 [63.2%] vs. 19 [50.0%]; 7.5 mm: 14 [36.8%] vs. 19 [50.0%]).

## Data Availability

Individual participant data are not publicly available, in line with the trial registration; de-identified participant-level data, the data dictionary, and the analysis syntax may be shared with qualified researchers upon reasonable request to the corresponding author, subject to ethics committee approval. The trial registration record (ClinicalTrials.gov NCT07148024) is provided as Supplementary File S1 and Supplementary Tables S1–S9 accompany this article.

## Supplementary Materials

The following supporting information can be downloaded at: https://www.mdpi.com/article/10.3390/medicina62101829/s1, Table S1: Estimated marginal means (GEE, exchangeable, robust SE) and between-group differences at each time point; Table S2: Group × time interaction coefficients (β, 95% CI; reference: 0 cmH2O group, T0); Table S3: Sensitivity of the T2 interaction coefficient and the interaction Wald χ^2^ to the working correlation structure; Table S4: Standardised mean differences for baseline characteristics (post hoc); Table S5: Adherence to cuff-pressure protocol during CPB; Table S6: Sensitivity analysis with the pre-intervention time point (T1) as baseline: GEE group × time interaction coefficients for T2 and T3 relative to T1 (exchangeable working correlation, robust SE); Table S7: Within-group sequential and baseline-referenced changes in ultrasonographic parameters (paired analysis, Wilcoxon signed-rank test); Table S8: Separate sensitivity analyses of the GEE group × time interaction with adjustment for Mallampati class or for CPB duration; Table S9: Dependence of the cuff-pressure effect on CPB duration (post hoc); File S1: ClinicalTrials.gov trial registration record (NCT07148024).
