# Supplementary material for: Point-of-Care Ultrasound of Upper Airway Changes During Cardiopulmonary Bypass in Coronary Artery Bypass Grafting: A Randomized Controlled Trial
Source: Medicina (Kaunas). 2026 Sep 22;62(10):1829. doi: 10.3390/medicina62101829 (PMC13706700; doi:10.3390/medicina62101829)
Supplement: Supplementary file 1 [file medicina-62-01829-s001.zip › medicina-4481034 - File S1.pdf]

ClinicalTrials.gov Protocol Registration and Results System (PRS) Receipt
Release Date: July 11, 2026

ClinicalTrials.gov ID: NCT07148024

Study Identification

Unique Protocol ID: KSH-ANREA-EC-03

Brief Title: Cuff Pressure and Airway Edema in CABG With CPB

Official Title: Effects of Endotracheal Tube Cuff Pressure Management on Upper Airway Edema During Cardiopulmonary Bypass in Coronary Artery Bypass Grafting: A Double-Blind, Randomized Controlled Trial

Secondary IDs:

Study Status

Record Verification: July 2026

Overall Status: Completed

Study Start: September 24, 2025 [Actual]

Primary Completion: April 19, 2026 [Actual]

Study Completion: April 19, 2026 [Actual]

Sponsor/Collaborators

Sponsor: Engin Çetin

Responsible Party: Sponsor-Investigator
Investigator: Engin Çetin [ECetin]
Official Title: principal investigator
Affiliation: Kocaeli City Hospital

Collaborators:

Oversight

U.S. FDA-regulated Drug: No

U.S. FDA-regulated Device: No

U.S. FDA IND/IDE: No

Human Subjects Review: Board Status: Approved
Approval Number: KAEK/11.bI.09
Board Name: Republic of Türkiye Kocaeli University Rectorate Clinical Research Ethics Committee
Board Affiliation: Republic of Türkiye Kocaeli University Rectorate Clinical Research Ethics Committee
Phone: 2623037450
Email: etikkurul@kocaeli.edu.tr

Address:

Baki Konsuoglu boulevard Umuttepe Campus Izmit/Kocaeli

Data Monitoring: No

Study Description

Brief Summary: This study investigates how endotracheal tube (ETT) cuff pressure management during cardiopulmonary bypass (CPB) in coronary artery bypass grafting (CABG) affects upper airway edema. Patients will be randomized into two groups: cuff pressure kept at 0 mmHg or maintained at 20–30 mmHg during CPB. Ultrasonography will be used to measure lateral pharyngeal wall thickness, tongue parameters, and other airway dimensions at predefined perioperative time points. The primary outcome is the change in lateral pharyngeal wall thickness as an indicator of airway edema. A total of 76 patients aged 18–80 years undergoing elective CABG with CPB will be enrolled. The results aim to clarify safe cuff pressure management strategies to reduce airway edema.

Detailed Description: Purpose This study aims to evaluate the effect of endotracheal tube (ETT) cuff pressure management during cardiopulmonary bypass (CPB) in coronary artery bypass grafting (CABG) surgery on the development of upper airway edema. Ultrasonography will be used to assess changes in lateral pharyngeal wall (LPW) thickness, tongue thickness, correlation between intravenous fluid volume at multiple perioperative time points.

Study Design

This is a prospective, randomized, double-blind, controlled clinical trial. Patients undergoing elective CABG with CPB will be randomly assigned into two groups:

Group 1: ETT cuff pressure maintained at 0 mmHg during CPB Group 2: ETT cuff pressure maintained at 20–30 mmHg during CPB Cuff pressures will be monitored with a manometer every 5 minutes and adjusted as needed. At the end of CPB, all patients will have standard cuff pressure (20–30 mmHg).

Outcomes Primary outcome: Changes in LPW thickness as a marker of upper airway edema assessed by ultrasonography at five time points (T0: Preoperative T1: Before initiation of CPB T2: At the end of CPB T3: 2nd postoperative hour).

Secondary outcomes: Changes in tongue thickness, correlation between intravenous fluid volume and airway edema.

Participation Approximately 76 patients (18–80 years, ASA III–IV) scheduled for elective CABG with CPB will be enrolled.

Conditions

Conditions: Airway Ultrasound
Airway Edema
Endotracheal Tube

Keywords: airway edema
coronary Bypass Greft Surgery
endotrecheal tube

Study Design

Study Type: Interventional

Primary Purpose: Diagnostic

Study Phase: N/A

Interventional Study Model: Parallel Assignment
Parallel assigment

Number of Arms: 2

Masking: Double (Participant, Outcomes Assessor)

Allocation: Randomized

Enrollment: 76 [Actual]

Arms and Interventions

Arms	Assigned Interventions
Active Comparator: ETT cuff pressure 0 mmHg<br>ETT cuff pressure will be set to 0 mmHg	Device: ETT cuff pressure 0 mmHg<br>In this group, the endotracheal tube (ETT) cuff pressure will be maintained at 0 mmHg during cardiopulmonary bypass (CPB)
Active Comparator: ETT cuff pressure 20-30 mmHg<br>ETT cuff pressure will be set to 20-30 mmHg	Device: ETT cuff pressure 20-30 mmHg<br>In this group, the endotracheal tube (ETT) cuff pressure will be maintained at 20-30 mmHg during cardiopulmonary bypass (CPB)

Outcome Measures

Primary Outcome Measure:

1. Change in Lateral Pharyngeal Wall Thickness Measured by Airway Ultrasound
   To compare the effect of two different endotracheal tube (ETT) cuff pressures (0 mmHg vs. 20–30 mmHg) during cardiopulmonary bypass (CPB) in coronary artery bypass grafting (CABG) on upper airway edema. The degree of airway edema will be assessed by measuring changes in lateral pharyngeal wall thickness (in millimeters, mm) using ultrasound at predefined perioperative time points.

   [Time Frame: T0: Preoperative T1: Before initiation of CPB T2: At the end of CPB T3: 2nd postoperative hour]

Secondary Outcome Measure:

2. ultrasonographic airway parameter -tongue width
   To compare the effects of two different endotracheal tube (ETT) cuff pressures (0 mmHg vs. 20–30 mmHg) during cardiopulmonary bypass (CPB) in coronary artery bypass grafting (CABG) on upper airway edema. The degree of edema will be assessed by changes in ultrasonographic airway parameter, specifically tongue width (mm), measured before and after the procedure

   [Time Frame: T0: Preoperative T1: Before initiation of CPB T2: At the end of CPB T3: 2nd postoperative hour]

3. ultrasonographic airway parameter -tongue volume
   To compare the effects of two different endotracheal tube (ETT) cuff pressures (0 mmHg vs. 20–30 mmHg) during cardiopulmonary bypass (CPB) in coronary artery bypass grafting (CABG) on the development of upper airway edema, which will be assessed by ultrasonographic measurements of upper airway structures before and after the procedure, including tongue thickness (mm).Tongue thickness (millimeters, mm)

   [Time Frame: T0: Preoperative T1: Before initiation of CPB T2: At the end of CPB T3: 2nd postoperative hour]

4. Correlation Between Intravenous Fluid Volume and Airway Edema

To evaluate the correlation between the total intravenous fluid volume administered intraoperatively and the degree of airway edema, as measured by changes in ultrasonographic airway parameters (e.g., tongue thickness, pharyngeal wall thickness) at defined time points.

[Time Frame: T0: Preoperative T1: Before initiation of CPB T2: At the end of CPB T3: 2nd postoperative hour]

Eligibility

Minimum Age: 18 Years

Maximum Age: 80 Years

Sex: All

Gender Based: No

Accepts Healthy Volunteers: No

Criteria: Inclusion Criteria:

- Patients scheduled for elective coronary artery bypass grafting (CABG) surgery
- Aged 18–80 years
- ASA physical status class III–IV
- Providing written informed consent

Exclusion Criteria:

- History of difficult intubation
- Anatomical abnormalities of the upper airway
- Body mass index (BMI) > 35 kg/m²
- Acute respiratory tract infection
- Emergency surgeries

Contacts/Locations

Central Contact Person: Engin ÇETİN
Telephone: 5321206004 Ext. 90
Email: Doccetin52@gmail.com

Central Contact Backup: Engin Çetin
Telephone: 05321206004
Email: Doccetin52@gmail.com

Study Officials: Engin Çetin
Study Principal Investigator
Kocaeli City Hospital

Locations: **Turkey (Türkiye)**
University of Health Sciences Kocaeli City Hospital
Kocaeli, Turkey (Türkiye)
Contact: MEHMET YILMAZ 505 217 44 32 Ext. 90
drmyilmaz33@gmail.com

IPDSharing

Plan to Share IPD: No
NO

References

Citations: Aytac BG, Soyal OB. Ultrasonographic evaluation of the postoperative airway edema after robotic prostatectomy: a single center observational study. Eur Rev Med Pharmacol Sci. 2023 Sep;27(18):8505-8513. doi: 10.26355/eurrev_202309_33775. PubMed 37782166

Links:

Available IPD/Information:

U.S. National Library of Medicine | U.S. National Institutes of Health | U.S. Department of Health & Human Services
