# Supplementary material for: Point-of-Care Ultrasound of Upper Airway Changes During Cardiopulmonary Bypass in Coronary Artery Bypass Grafting: A Randomized Controlled Trial
Source: Medicina (Kaunas). 2026 Sep 22;62(10):1829. doi: 10.3390/medicina62101829 (PMC13706700; doi:10.3390/medicina62101829)
Supplement: Supplementary file 1 [file medicina-62-01829-s001.zip › medicina-4481034-supplementary.pdf]

Supplementary Material

Point-of-Care Ultrasound of Upper Airway Changes During Cardiopulmonary Bypass in Coronary Artery Bypass Grafting: A Randomized Controlled Trial (Manuscript ID: medicina-4481034)

Supplementary Tables S1–S9. Supplementary File S1 (ClinicalTrials.gov registration record NCT07148024) is provided as a separate PDF. Classification of analyses: S1–S3, supporting analyses of the primary and secondary ultrasonographic outcomes; S4, post hoc; S5, protocol adherence; S6, sensitivity analysis; S7, supporting within-group analyses; S8, post hoc sensitivity analyses; S9, post hoc exploratory analysis.

Supplementary Table S1. Estimated marginal means (GEE, exchangeable, robust SE) and between-group differences at each time point

Parameter	Time	0 cmH2O, mean (95% CI)	20–30 cmH2O, mean (95% CI)	Difference 20–30 minus 0 (95% CI)	p
LPW thickness (mm)	T0	10.94 (10.81 to 11.07)	10.98 (10.87 to 11.10)	0.04 (-0.13 to 0.21)	0.632
	T1	11.15 (11.01 to 11.29)	11.26 (11.11 to 11.41)	0.11 (-0.09 to 0.31)	0.297
	T2	12.24 (12.09 to 12.40)	13.79 (13.57 to 14.00)	1.54 (1.28 to 1.81)	<0.001
	T3	11.90 (11.74 to 12.05)	12.94 (12.76 to 13.12)	1.04 (0.80 to 1.28)	<0.001
Parapharyngeal distance (mm)	T0	15.09 (14.84 to 15.34)	15.23 (14.96 to 15.50)	0.14 (-0.22 to 0.50)	0.453
	T1	15.38 (15.11 to 15.64)	15.33 (15.02 to 15.63)	-0.05 (-0.45 to 0.36)	0.819
	T2	16.21 (15.89 to 16.52)	17.95 (17.54 to 18.36)	1.75 (1.23 to 2.27)	<0.001
	T3	15.93 (15.63 to 16.23)	16.99 (16.63 to 17.34)	1.06 (0.59 to 1.52)	<0.001
Tongue thickness (mm)	T0	55.81 (55.31 to 56.32)	55.83 (55.44 to 56.21)	0.01 (-0.62 to 0.65)	0.968
	T1	56.13 (55.62 to 56.64)	56.78 (56.38 to 57.19)	0.66 (0.01 to 1.31)	0.048
	T2	56.88 (56.36 to 57.40)	58.23 (57.78 to 58.68)	1.36 (0.67 to 2.04)	<0.001
	T3	56.73 (56.21 to 57.25)	57.75 (57.33 to 58.17)	1.02 (0.35 to 1.69)	0.003
Tongue width (mm)	T0	48.07 (47.81 to 48.33)	48.00 (47.68 to 48.32)	-0.07 (-0.48 to 0.34)	0.736
	T1	48.20 (47.92 to 48.47)	48.29 (47.97 to 48.62)	0.09 (-0.33 to 0.52)	0.664
	T2	48.39 (48.10 to 48.68)	48.88 (48.52 to 49.23)	0.49 (0.03 to 0.94)	0.037
	T3	48.28 (48.00 to 48.56)	48.67 (48.33 to 49.02)	0.39 (-0.06 to 0.84)	0.086
Midsagittal tongue area (cm^2)	T0	25.64 (25.27 to 26.00)	25.84 (25.46 to 26.21)	0.20 (-0.32 to 0.72)	0.450
	T1	25.28 (24.91 to 25.66)	25.17 (24.79 to 25.55)	-0.11 (-0.65 to 0.42)	0.678
	T2	24.62 (24.19 to 25.06)	23.83 (23.43 to 24.23)	-0.79 (-1.38 to -0.20)	0.009
	T3	24.84 (24.44 to 25.25)	24.35 (23.98 to 24.73)	-0.49 (-1.05 to 0.06)	0.082

Unadjusted p-values from the robust Wald test of the time-point contrast. T0 pre-intubation; T1 post-intubation, pre-CPB; T2 post-CPB; T3 post-operative hour 2. Bonferroni-adjusted p-values for the between-group comparison at each time point (28 pairwise comparisons of the eight group × time estimated marginal means) are given in Table 3 of the main text; these time-point comparisons are supporting analyses (LPW) or exploratory follow-up comparisons (secondary parameters).

Supplementary Table S2. Group × time interaction coefficients (β, 95% CI; reference: 0 cmH2O group, T0)

Parameter	β T1 (95% CI)	β T2 (95% CI) – supporting contrast for LPW	β T3 (95% CI)	Wald χ² (interaction, 3 df)	Working correlation α
LPW thickness (mm)	0.07 (-0.06 to 0.19)	1.50 (1.27 to 1.73)	1.00 (0.82 to 1.18)	665.6	0.76
Parapharyngeal distance (mm)	-0.19 (-0.31 to -0.07)	1.61 (1.30 to 1.92)	0.92 (0.70 to 1.13)	685.1	0.89
Tongue thickness (mm)	0.64 (0.53 to 0.76)	1.34 (1.06 to 1.62)	1.01 (0.77 to 1.24)	126.1	0.95
Tongue width (mm)	0.17 (0.08 to 0.25)	0.56 (0.38 to 0.73)	0.46 (0.31 to 0.61)	42.2	0.95
Midsagittal tongue area (cm²)	-0.31 (-0.44 to -0.19)	-0.99 (-1.32 to -0.67)	-0.69 (-0.95 to -0.43)	37.9	0.92

Supplementary Table S3. Sensitivity of the T2 interaction coefficient and the interaction Wald χ² to the working correlation structure

Parameter	Exchangeable β / χ²	AR(1) β / χ²	Unstructured β / χ²	Independence β / χ²
LPW thickness (mm)	1.50 / 665.6	1.50 / 665.6	1.50 / 665.6	1.50 / 665.6
Parapharyngeal distance (mm)	1.61 / 685.1	1.61 / 685.1	1.61 / 685.1	1.61 / 685.1
Tongue thickness (mm)	1.34 / 126.1	1.34 / 126.1	1.34 / 126.1	1.34 / 126.1
Tongue width (mm)	0.56 / 42.2	0.56 / 42.2	0.56 / 42.2	0.56 / 42.2
Midsagittal tongue area (cm²)	-0.99 / 37.9	-0.99 / 37.9	-0.99 / 37.9	-0.99 / 37.9

Estimates and robust standard errors are identical to the reported precision across structures, as expected for a saturated mean model with complete, balanced data.

Supplementary Table S4. Standardised mean differences for baseline characteristics (post hoc)

Variable	SMD (0 minus 20–30 cmH2O)
Age (years)	−0.07
Body mass index	−0.05
Male sex	0.27
ASA IV	0.07
Mallampati I	0.54
Mallampati II	−0.37
Mallampati III	−0.06
Mallampati IV	0.11

Supplementary Table S5. Adherence to cuff-pressure protocol during CPB

Group	Checks	Recorded cuff pressure, cmH2O	Readings outside target	Adjustments
0 cmH2O (n = 38)	Every 5 min	0 (fully deflated) at all checks	0	Not applicable
20–30 cmH2O (n = 38)	Every 5 min	Mean 24.7 ± 3.3; range 20–30	0	Not recorded

Supplementary Table S6. Sensitivity analysis with the pre-intervention time point (T1) as baseline: GEE group × time interaction coefficients for T2 and T3 relative to T1 (exchangeable working correlation, robust SE)

Parameter	Difference in change T1→T2, 20–30 minus 0 cmH2O (95% CI)	Difference in change T1→T3 (95% CI)	Wald χ^2 (interaction, 2 df)	p
LPW thickness (mm)	1.43 (1.30 to 1.57)	0.93 (0.83 to 1.04)	442.9	<0.001
Parapharyngeal distance (mm)	1.79 (1.58 to 2.01)	1.10 (0.96 to 1.24)	279.0	<0.001
Tongue thickness (mm)	0.70 (0.51 to 0.89)	0.36 (0.20 to 0.52)	86.0	<0.001
Tongue width (mm)	0.39 (0.27 to 0.52)	0.30 (0.19 to 0.41)	38.6	<0.001
Midsagittal tongue area (cm^2)	-0.68 (-0.91 to -0.45)	-0.38 (-0.54 to -0.22)	37.0	<0.001

Models fitted to T1–T3 only, with T1 as reference; coefficients are the between-group differences in change from T1.

Manuscript ID: medicina-4481034 — Point-of-Care Ultrasound of Upper Airway Changes During Cardiopulmonary Bypass in Coronary Artery Bypass Grafting: A Randomized Controlled Trial

Supplementary Tables S7–S9

Supplementary Table S7. Within-group sequential and baseline-referenced changes in ultrasonographic parameters (paired analysis, Wilcoxon signed-rank test).

Parameter	Contrast	Group 0 cmH_2O: change (% of earlier value)	p	Group 20–30 cmH_2O: change (% of earlier value)	p
LPW	T1–T0	+0.21 ± 0.23 (+1.9%)	<0.001	+0.28 ± 0.31 (+2.5%)	<0.001
LPW	T2–T1	+1.09 ± 0.26 (+9.8%)	<0.001	+2.53 ± 0.34 (+22.4%)	<0.001
LPW	T3–T2	-0.35 ± 0.18 (-2.8%)	<0.001	-0.85 ± 0.23 (-6.1%)	<0.001
LPW	T2–T0	+1.30 ± 0.42 (+12.0%)	<0.001	+2.80 ± 0.60 (+25.5%)	<0.001
LPW	T3–T0	+0.96 ± 0.37 (+8.8%)	<0.001	+1.96 ± 0.45 (+17.8%)	<0.001
Parapharyngeal	T1–T0	+0.28 ± 0.18 (+1.9%)	<0.001	+0.10 ± 0.34 (+0.6%)	0.120
Parapharyngeal	T2–T1	+0.83 ± 0.38 (+5.4%)	<0.001	+2.62 ± 0.57 (+17.1%)	<0.001
Parapharyngeal	T3–T2	-0.27 ± 0.18 (-1.7%)	<0.001	-0.97 ± 0.35 (-5.3%)	<0.001
Parapharyngeal	T2–T0	+1.11 ± 0.46 (+7.4%)	<0.001	+2.72 ± 0.87 (+17.9%)	<0.001
Parapharyngeal	T3–T0	+0.84 ± 0.37 (+5.5%)	<0.001	+1.76 ± 0.58 (+11.5%)	<0.001
Tongue thickness	T1–T0	+0.31 ± 0.18 (+0.6%)	<0.001	+0.96 ± 0.31 (+1.7%)	<0.001
Tongue thickness	T2–T1	+0.75 ± 0.41 (+1.3%)	<0.001	+1.45 ± 0.44 (+2.5%)	<0.001
Tongue thickness	T3–T2	-0.14 ± 0.14 (-0.3%)	<0.001	-0.48 ± 0.19 (-0.8%)	<0.001

Parameter	Contrast	Group 0 cmH₂O: change (% of earlier value)	p	Group 20–30 cmH₂O: change (% of earlier value)	p
Tongue thickness	T2–T0	+1.06 ± 0.53 (+1.9%)	<0.001	+2.41 ± 0.71 (+4.3%)	<0.001
Tongue thickness	T3–T0	+0.92 ± 0.47 (+1.6%)	<0.001	+1.93 ± 0.58 (+3.5%)	<0.001
Tongue width	T1–T0	+0.13 ± 0.18 (+0.3%)	<0.001	+0.29 ± 0.20 (+0.6%)	<0.001
Tongue width	T2–T1	+0.19 ± 0.28 (+0.4%)	<0.001	+0.58 ± 0.29 (+1.2%)	<0.001
Tongue width	T3–T2	-0.11 ± 0.17 (-0.2%)	<0.001	-0.20 ± 0.20 (-0.4%)	<0.001
Tongue width	T2–T0	+0.32 ± 0.37 (+0.7%)	<0.001	+0.88 ± 0.43 (+1.8%)	<0.001
Tongue width	T3–T0	+0.21 ± 0.32 (+0.4%)	<0.001	+0.68 ± 0.35 (+1.4%)	<0.001
Midsagittal area	T1–T0	-0.35 ± 0.23 (-1.4%)	<0.001	-0.67 ± 0.32 (-2.6%)	<0.001
Midsagittal area	T2–T1	-0.66 ± 0.43 (-2.6%)	<0.001	-1.34 ± 0.58 (-5.3%)	<0.001
Midsagittal area	T3–T2	+0.22 ± 0.23 (+0.9%)	<0.001	+0.52 ± 0.25 (+2.2%)	<0.001
Midsagittal area	T2–T0	-1.01 ± 0.59 (-4.0%)	<0.001	-2.01 ± 0.85 (-7.7%)	<0.001
Midsagittal area	T3–T0	-0.79 ± 0.50 (-3.1%)	<0.001	-1.48 ± 0.65 (-5.7%)	<0.001

Values are mean ± SD of the within-patient difference (later minus earlier time point) with the mean percentage change relative to the earlier time point; p-values from the Wilcoxon signed-rank test (n = 38 per group). Units: LPW, parapharyngeal distance, tongue thickness and tongue width in mm; midsagittal tongue area in cm². T0, before induction; T1, after intubation before CPB; T2, end of CPB; T3, postoperative hour 2. These are supporting analyses; the primary test is the GEE group × time interaction (Table 4).

Supplementary Table S8. Separate sensitivity analyses of the GEE group × time interaction with adjustment for Mallampati class or for CPB duration.

Parameter	Model	Group × time Wald χ^2 (3 df)	p	Group × time coefficient at T2, mm or cm² (95% CI)	Group × time coefficient at T3 (95% CI)
LPW	Unadjusted	665.6	<0.001	1.50 (1.27 to 1.73)	1.00 (0.82 to 1.18)
LPW	+Mallampati × time	660.5	<0.001	1.49 (1.25 to 1.72)	0.98 (0.80 to 1.17)
LPW	+CPB duration × time	712.3	<0.001	1.56 (1.33 to 1.78)	1.03 (0.85 to 1.22)
Parapharyngeal	Unadjusted	685.1	<0.001	1.61 (1.30 to 1.92)	0.92 (0.70 to 1.13)
Parapharyngeal	+Mallampati × time	602.8	<0.001	1.62 (1.29 to 1.95)	0.92 (0.70 to 1.15)
Parapharyngeal	+CPB duration × time	731.1	<0.001	1.69 (1.40 to 1.98)	0.97 (0.77 to 1.18)
Tongue thickness	Unadjusted	126.1	<0.001	1.34 (1.06 to 1.62)	1.01 (0.77 to 1.24)
Tongue thickness	+Mallampati × time	136.5	<0.001	1.33 (1.06 to 1.60)	0.99 (0.76 to 1.22)
Tongue thickness	+CPB duration × time	173.6	<0.001	1.45 (1.21 to 1.68)	1.09 (0.89 to 1.29)
Tongue width	Unadjusted	42.2	<0.001	0.56 (0.38 to 0.73)	0.46 (0.31 to 0.61)
Tongue width	+Mallampati × time	37.5	<0.001	0.57 (0.37 to 0.76)	0.47 (0.31 to 0.63)
Tongue width	+CPB duration × time	42.0	<0.001	0.57 (0.39 to 0.75)	0.48 (0.33 to 0.63)
Midsagittal area	Unadjusted	37.9	<0.001	-0.99 (-1.32 to -0.67)	-0.69 (-0.95 to -0.43)
Midsagittal area	+Mallampati × time	32.0	<0.001	-1.07 (-1.46 to -0.69)	-0.76 (-1.06 to -0.45)

Parameter	Model	Group × time Wald χ^2 (3 df)	p	Group × time coefficient at T2, mm or cm² (95% CI)	Group × time coefficient at T3 (95% CI)
Midsagittal area	+CPB duration × time	47.2	<0.001	-1.07 (-1.38 to -0.76)	-0.76 (-1.00 to -0.52)

Linear GEE (identity link, exchangeable working correlation, robust SE), patient as cluster. "Unadjusted" reproduces Table 4. "+Mallampati × time" adds Mallampati class (I–IV) and its interaction with time; "+CPB duration × time" adds CPB duration (centred, min) and its interaction with time. Coefficients are the between-group differences (20–30 minus 0 cmH_2O) in change from T0. Adding Mallampati class as a main effect only leaves the interaction coefficients identical to the unadjusted model (saturated mean model). Post hoc Mallampati-stratified analysis of difficult extubation: Cochran–Mantel–Haenszel χ^2 = 10.5, p = 0.001 (strata I vs II–IV).

Supplementary Table S9. Dependence of the cuff-pressure effect on CPB duration (post hoc).

Parameter	Group × CPB duration × time Wald χ^2 (3 df)	p	Three-way coefficient at T2 per 10 min of CPB (95% CI)	Spearman ρ, Δ(T2–T0) vs CPB duration, 0 cmH_2O (p)	Spearman ρ, 20–30 cmH_2O (p)
LPW	1.50	0.682	-0.026 (-0.198 to 0.147)	0.28 (0.088)	0.23 (0.158)
Tongue width	5.72	0.126	-0.039 (-0.197 to 0.119)	0.11 (0.527)	0.08 (0.652)
Midsagittal area	9.12	0.028	-0.366 (-0.613 to -0.119)	-0.04 (0.820)	-0.33 (0.040)
Tongue thickness	3.09	0.378	0.056 (-0.141 to 0.252)	0.52 (<0.001)	0.31 (0.058)
Parapharyngeal	13.35	0.004	0.287 (0.104 to 0.470)	0.14 (0.418)	0.51 (0.001)

The three-way coefficient is the change in the between-group difference (20–30 minus 0 cmH_2O) in the T0→T2 change per 10 min of CPB. CPB duration ranged from 70 to 127 min. These analyses were not pre-specified and are exploratory.
